# rhCNB Improves Cyclophosphamide-Induced Immunodeficiency in BALB/c Mice

**DOI:** 10.1155/2022/4891399

**Published:** 2022-09-27

**Authors:** Wenhua Zhong, Hui Huang, Zhaoxin Yang, Penghuan Chang

**Affiliations:** ^1^College of Pharmacy, Hainan Medical University, Haikou 571199, China; ^2^Department of Nursing, Haikou People's Hospital, Affiliated Haikou Hospital of Central South University Xiangya School of Medicine, Haikou 570100, China

## Abstract

**Background:**

This study aims to explore the immunomodulatory effect of rhCNB on mice with cyclophosphamide (CTX)-induced immunodeficiency through TLR_4_/MAPK pathway.

**Methods:**

BALB/c mice were randomly divided into three groups: a negative control group, an immunosuppression model group, and a rhCNB treatment group. Tail vein injection of cyclophosphamide (40 mg/kg) was used to establish a mouse immunosuppression model. Intraperitoneal injection of rhCNB (20 mg/kg) was administered to the treatment group, whereas equal quantities of normal saline were given to the control group and model group. Perform peripheral blood routine of CD4, CD8, and CD19 lymphocyte subsets and peripheral blood Th1/Th2 cell subsets 24 hours after the last administration. RT-PCR was used to detect mRNA levels of TLR_4_, P38, JNK, T-bet, and GATA_3_, the spleen immune organ index was measured, and the histopathological status of the spleen and thymus was observed.

**Results:**

The results showed that compared with the control group, WBC, PLT, LYM, NEU, immune organ index, CD4^+^/CD8^+^ and CD19^+^ subgroup ratio, and peripheral blood Th1/Th2 cell subgroups decreased in the model group. The mRNA levels of TLR_4_, P38, JNK, T-bet, and GATA_3_ decreased compared with the model group, while they increased in the treatment group.

**Conclusions:**

rhCNB has an immunomodulatory effect by regulating the expression of Th1/Th2 cytokine balance through the TLR_4_/MAPK signaling pathway and promoting the differentiation and proliferation of lymphocytes, thereby improving the immune function.

## 1. Introduction

A malignant tumor is one of the major diseases threatening human life and health, and its mortality rate is second only to cardiovascular diseases [1–3]. Currently, the main treatments for malignant tumors are surgery, drug chemotherapy, radiotherapy, and chemotherapy. The body's bone marrow hematopoietic system and immune function are impaired when cancer cells are killed by radiation or drugs, which causes bone marrow suppression and a reduction in immune function. This would not only lower the quality of life for patients but also further reduce the effectiveness of the treatment, so one of cancer treatment goals is to lessen the influence on the body's immune system.

The recombinant human calcineurin B subunit (rhCNB) is a protein obtained by constructing the recombinant strain with high expression. TLR_4_/NF-*κ*B signaling pathway can activate the innate immune system, promote dendritic cell maturation, upregulate the expression of macrophages, and cause the production of TNF-*α*, IL-6, IL-12, IL-1*β*, and CCL_5_, as well as mediate tumor cell apoptosis [4–7].

As a membrane recognition receptor protein, TLR is an important barrier against infectious diseases. The most common example is toll-like receptor 4 (TLR_4_), which not only activates the genes related to innate immunity and adaptive immune response but also promotes the release of related cytokines to regulate immune function [8, 9]. Activated TLR_4_ can activate the mitogen-activated protein kinase (MAPK) signaling pathway downstream [10]. The MAPK signaling pathway contributes to cell proliferation, differentiation, and adaptation to environmental stress [11]. Through the TLR_4_ pathway, rhCNB can activate macrophages, promote dendritic maturation, enhance the antigen-presenting effect of antigen-presenting cells, and promote activity of phagocytic and NK cells in macrophages. Besides, rhCNB induces the secretion of proinflammatory cytokines and chemokines, enhances cellular immunity, and causes apoptosis of tumor cells [7]. This study investigated how rhCNB affects the immune function of mice with cyclophosphamide (CTX)-induced immunodeficiency. The changes in TLR_4_ and Th1/Th2 level and MAPK signal pathway were detected at the mRNA level to determine whether rhCNB regulates the immune function of immunosuppressed mice through TLR_4_/MAPK pathway. The present research aims to provide reference for the further study of rhCNB in regulating immunologic function and related pharmacology after radiotherapy and chemotherapy.

## 2. Materials and Methods

### 2.1. Animals and Experimental Design

Female BALB/c mice (Changsha Tianqin Biotechnology Co., Changsha, China) aged six to eight weeks, weighing 18–22 g, were housed in polypropylene cages in specific pathogen-free rooms kept at 20–26°C with 40–70% relative humidity and a 12 h light cycle. All animals were given free access to standard rodent chow and filtered tap water *ad libitum*. All experiments were approved by and proceeded following the Hainan Medical College Animal Care and Use Committee (HYLL-2021-148).

The mice were randomly divided into the control group, the CTX group (40 mg/kg) [12], and the rhCNB group (20 mg/kg). The CTX group and rhCNB group were injected with 40 mg/kg CTX through tail veins for 3 days, while the control group received an equivalent volume of saline injection. On the 4^th^ day after the model was established, the rhCNB group received an intraperitoneal injection of rhCNB once a day for 5 days. Saline was administered intraperitoneally to both the CTX and control groups.

### 2.2. Reagents

Anti-mCD4-APC (553051), anti-mCD3e-PerCP (553067), anti-mCD8-PE (553032), anti-mCD4-PerCP (550954), anti-mIFN-*γ*-APC (554413), and anti-mIL-4-PE (554435) were purchased from BD Biosciences (Franklin Lakes, NJ, US). PrimeScriptTMRT reagent kit and MiniBEST Universal RNA Extraction kit were purchased from Takara Bio. Cyclophosphamide was obtained from Jiangsu Baxter Oncology Gmbh (Kantstr, Halle, Germany). rhCNB is a gift from Haikou Qili Pharmaceutical Co. Ltd. (Haikou, China).

### 2.3. Spleen Organ Index

The mice were weighed 24 hours following the last treatment, and then spleens of each mouse were collected and weighed immediately to determine the immune organ index.

### 2.4. HE Staining of Spleen and Thymus Tissue

Spleen and thymus tissue were isolated from mice, fixed in 4% paraformaldehyde, embedded in paraffin, and cut into sections of 3 *µ*m. Following HE staining and sealing, these sections were examined under an optical microscope.

### 2.5. Hematological Examination

Blood was collected into EDTA-2K tubes by a retro-orbital bleeding method 24 hours after the last dose. Blood counts were performed using a hematology analyzer (ADVIA 2120i, Siemens, Germany), including white (WBC) and red blood cell (RBC) counts, platelet counts (PLT), lymphocyte percentage (LY%), and neutrophil ratio (NE%).

### 2.6. T-Lymphocyte and B-Lymphocyte Assay by Flow Cytometry

After being treated with RBC lysing buffer and PBS washing, the peripheral blood samples were incubated with anti-mouse CD3/CD4/CD8/CD19 antibodies (5 L) at 4°C for 1 hour. Utilizing BD FACS CaliburTM, flow cytometry analyses were carried out on the cells after they had been washed and resuspended in PBS (BD Biosciences, USA).

### 2.7. Detection of CD4^+^ IFN-*γ*^+^ and CD4^+^ IL-4^+^ Lymphocytes in Peripheral Blood by Flow Cytometry

Lymphocytes were separated from the peripheral blood of mice by a lymphocyte separation medium. Then, the cells in each tube were treated with Leuko Act Cktl with GolgiPlug (BD, 550583), and they were incubated for 4 hours at 37°C in an incubator with 5% CO_2_. After stimulation, the cells were incubated with PerCP-anti-CD4 for 30 minutes at room temperature in the dark, washed, and then mixed with fixation/permeabilization solution (BD, 554714) to incubate for another 30 minutes at room temperature in the dark. After being washed, the samples were incubated for 30 minutes with anti-IL-4-PE and anti-IFN-*γ*-APC, then washed, and resuspended with 300 *µ*L staining buffer. Samples were measured by flow cytometry.

### 2.8. Real-Time PCR

After tissues were subjected to Trizol reagent to isolate the quantitative real-time PCR total RNA, PrimeScriptTMRT reagent Kit (Takara Bio) was used to reverse-transcribe the RNA into cDNA. The cDNA was amplified by the MiniBEST Universal RNA Extraction Kit on a StepOnePlus Real-Time PCR System (Thermo Fisher Scientific). Primers are shown in Table 1. GAPDH was used as an internal control [13].

### 2.9. Statistical Analysis

Data are shown as mean ± SD. The statistical analysis program SPSS (v(0).24, SPSS Inc., Cary, NC) was used to determine the statistical significance of the differences between various groups using either Student's *t*-test or an ANOVA analysis for multiple comparisons. Values of *P* < 0.05 were considered statistically significant.

## 3. Results

### 3.1. Effect of rhCNB on Body Weight in Immunosuppressed Mice

**Figure 1:**
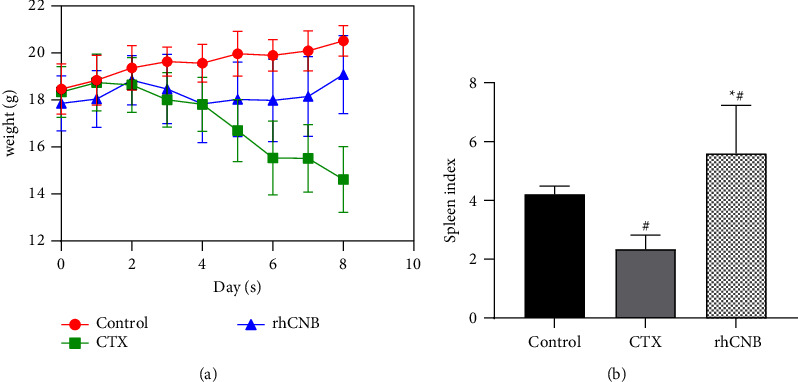
Body weight curve and spleen index. (a) The body weight curve. (b) The spleen index of mice (control is the negative control group, CTX is the model group, and rhCNB is the treatment group). Compared with control group, ^#^*P* < 0.05; compared with model group, ^*∗*^*P* < 0.05.

Compared with the control group, mice in the model group gradually lost weight after being given cyclophosphamide. After rhCNB intervention, the treatment group's body weight increased more slowly than that of the model group (Figure 1(a)). In addition, the mice in the model group showed upside-down coats, reduced mobility, and arched back after the administration of cyclophosphamide. After treatment with rhCNB, the mice in the treatment group had loose body hair, increased mobility, and reduced back phenomenon compared with the model group. It shows that rhCNB can reverse the weight loss induced by cyclophosphamide.

### 3.2. Effect of rhCNB on Immune Organ Index in Immunosuppressed Mice

The mice in the model group had significantly lower spleen index compared to the control group (*P* < 0.05). Compared with the model group, the spleen index of the mice in the treatment group increased, and the difference was statistically significant (*P* < 0.05). It indicates that rhCNB can significantly enhance the spleen index of immunosuppressed mice and increase the quality of the spleen (Figure 1(b)).

### 3.3. Effect of rhCNB on Histopathological Changes in Immunosuppressed Mice

**Figure 2:**
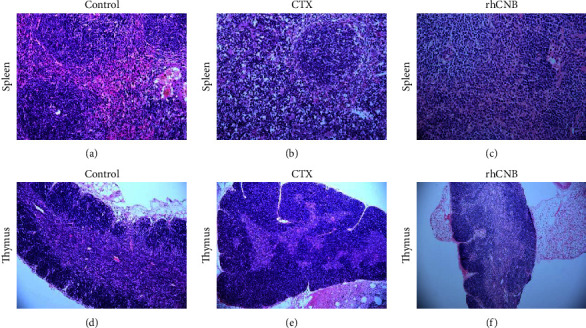
Effect of rhCNB on HE staining of the thymus (×200) and spleen (×400). (a, d) Normal tissue. (b) The spleen body shrinks, and the number of lymphocytes in the cap area decreases. (c) The volume of spleen corpuscle increased and the lymphocyte of cap area increased. (e) Glandular medulla area is partially reduced, the cortex increases, and the skin/medullary ratio increases. (f) The skin/medullary ratio decreases.

The spleen body size of the model group was smaller than that of the control group, the lymphocytes in the cap area were loosely arranged, and a small number of lymphocytes underwent apoptosis. After rhCNB treatment, the number of splenic corpuscles and tightly arranged lymphocytes in the cap area increased, and no apoptotic cells were observed. Compared with the control group, the thymus medulla part of the model group was reduced, and the cortex part and the cortex-medullary ratio increased. Cell necrosis/apoptosis of the starry sky phenomenon can be seen in the cortex. After rhCNB treatment, the medulla part increased, the cortex decreased, and the cortex-medullary ratio decreased. It demonstrates that rhCNB can reduce the damage to the spleen and thymus induced by cyclophosphamide and can restore the function of immune organs with reduced immune function (Figure 2).

### 3.4. Effect of rhCNB on the Hematological Index in Immunosuppressed Mice

**Figure 3:**
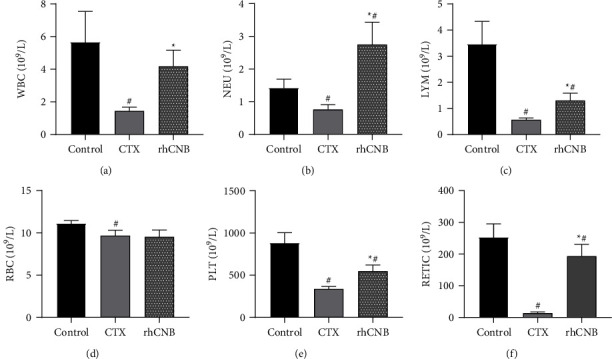
Analysis of rhCNB on hemogram of immunocompromised mice. The number of immune cells in the blood was analyzed to evaluate the immune function of mice.

Cyclophosphamide, a commonly used modeling drug for inducing immunosuppressive animal models, reduces the body's immune function and decreases the number of WBCs, LYM, NEU, and PLT [14, 15]. The number of WBCs, LYM, NEU, PLT, and RBCs in the CTX model group was significantly reduced (*P* < 0.05) compared with the control group but significantly increased (*P* < 0.05) in the rhCNB treatment group compared with the CTX model group. It shows that rhCNB can lessen the cyclophosphamide-induced bone marrow suppression and restore the body's hematopoietic function (Figure 3).

### 3.5. Effect of rhCNB on T-Lymphocyte Subsets and B-Lymphocyte Subsets

**Figure 4:**
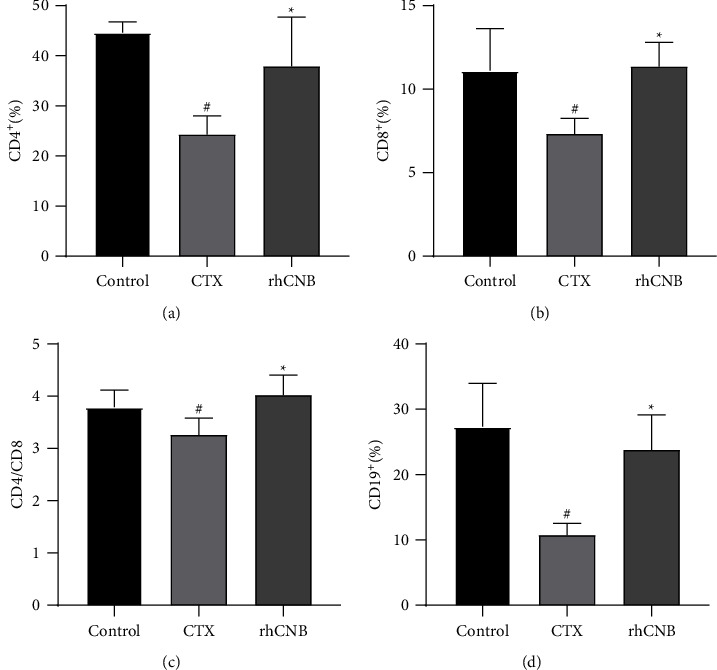
The results of peripheral blood lymphocyte subsets in each group. By analyzing the lymphatic subsets of T cells and B cells to evaluate the humoral and cellular immune functions of mice, it was found that rhCNB can improve the immune function of immunocompromised mice.

**Figure 5:**
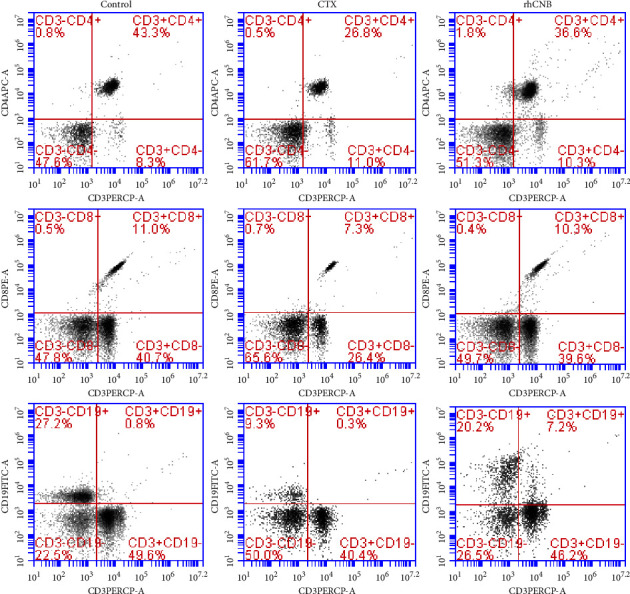
Examples of the detection results of peripheral blood lymphocyte subsets in each group. The flow cytometry was used to analyze the cell subsets, and the schematic diagram of the flow subsets in each group.

The number of CD4 and CD8 lymphocytes as well as the ratio of CD4^+^/CD8^+^ T cells dropped in the model group as compared to the control group (*P* < 0.05) but increased in the rhCNB treatment group compared with the model group (*P* < 0.05) (Figures 4(a)–4(c)). Compared with the control group, the number of CD19 lymphoid subgroups in the model group decreased (*P* < 0.05) but increased compared with the model group (*P* < 0.05) (Figure 4(d)). It proves that rhCNB can promote the differentiation and proliferation of T cell subgroups and B cell subgroups in immunocompromised mice and regulate and improve the body's immune response (Figure 5).

### 3.6. Effect of rhCNB on Th1/Th2 Balance in Peripheral Blood of Immunosuppressed Mice

**Figure 6:**
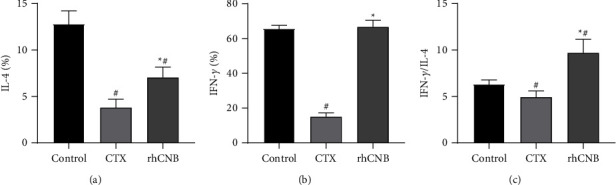
Results of flow cytometry IL-4 and IFN-*γ*. The effect of rhCNB on immune function was evaluated by analyzing the cytokines IFN-*γ* and IL-4 represented by Th1 and Th2. The results showed that rhCNB could restore the immune function of cyclophosphamide-induced immunocompromised mice.

The content of IL-4 and IFN-*γ* in the model group was reduced compared with the control group (*P* < 0.05) but increased in the rhCNB treatment group compared with the model group (*P* < 0.05) (Figures 6(a) and 6(b)). The ratio of IFN-*γ*/IL-4 in the model group decreased compared with the control group but increased in the rhCNB treatment group compared with the model group (*P* < 0.05) (Figure 6(c)). The results reveal that rhCNB can regulate the function of cell subsets in immunosuppressed mice by encouraging the release of Th1/Th2 cytokines and enhance the function of cellular and humoral immunity, thus regulating the state of low immunity.

### 3.7. The mRNA Expression Levels of TLR_4_, P38, JNK, ERK, T-Bet, and GATA_3_ in the TLR_4_/MAPK Signaling Pathway

**Figure 7:**
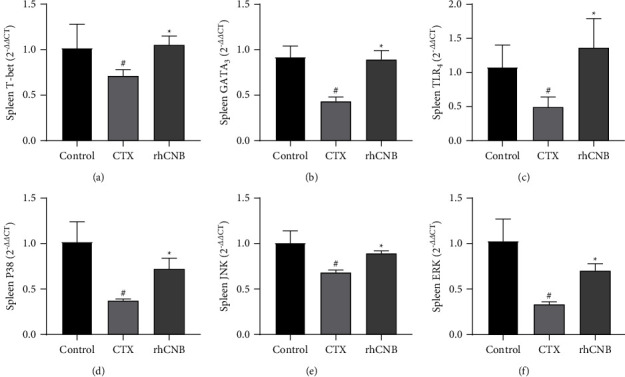
mRNA expression levels of related genes in spleen. The effect of rhCNB on the regulation of immune function was evaluated by PCR analysis of the spleen. The primer sequences are shown in Table 1. Data are shown as mean ± SD. The statistical analysis program SPSS was used to determine the statistical significance of the differences between various groups using either Student's *t*-test or an ANOVA analysis for multiple comparisons. Values of *P* < 0.05 were considered statistically significant.

The expression levels of TLR_4_, P38, JNK, and ERK in the model group decreased as compared to the control group (*P* < 0.05) but increased in the rhCNB treatment group compared with the model group (*P* < 0.05) (Figures 7(c)–7(f)). Compared with the control group, the expression levels of T-bet and GATA_3_ in the model group decreased (*P* < 0.05) but increased in the rhCNB treatment group compared with the model group (*P* < 0.05) (Figures 7(a) and 7(b)). It suggests that rhCNB can improve the immune function of mice by regulating Th1/Th2 levels through TLR_4_/MAPK signaling pathway.

## 4. Discussion

The current methods of treating malignant tumors are mainly surgery, drug chemotherapy, and radiotherapy. However, when tumor cells are killed by radiation and drugs, the body's bone marrow hematopoietic system and immune function will be damaged, which in turn leads to bone marrow suppression and reduces patients' immune function. As a result, patients' quality of life and treatment effect will be reduced. rhCNB is a protein drug with an anti-tumor effect, which can mediate tumor cell apoptosis by stimulating macrophages [16]. While rhCNB is an immunostimulatory protein, it has not yet been determined if it can ameliorate and regulate the immune suppression state of the body after radiotherapy and chemotherapy and restore immunological function in the body as a whole. Therefore, this study aims to investigate the effect of rhCNB on the immune system of mice with cyclophosphamide-induced immunodeficiency.

Although cyclophosphamide, a common cytotoxic agent, is frequently used to treat malignant tumors, it can cause atrophy of immune organs, weight loss, leukopenia, lymphopenia, bone marrow suppression, and thrombocytopenia and inhibit the humoral and cellular immunity of animals. Therefore, cyclophosphamide is often used as a model drug to replicate the animal model of immunosuppression and to study the immunomodulatory effect of drugs [17–21]. The spleen, the body's largest immune organ, is the body's cellular and humoral immune center. It can exert non-specific immune functions through phagocytosis and specific immune functions through T and B cell-mediated cellular and humoral immunity [22, 23]. This study observed the body weight of mice, the pathology of the spleen and thymus, the index of immune organs, and the blood cell counts. The results showed that rhCNB could significantly increase the immune organ index of immunosuppressed mice, increase the quality of spleen organs, and recover the weight loss induced by cyclophosphamide. The thymus and spleen are important immune organs to maintain immune homeostasis. The pathological results of the spleen and thymus indicated that rhCNB could recover the injury of the spleen and thymus induced by cyclophosphamide. The reduction of WBCs is the most common complication after chemotherapy. WBCs are considered to be mainly related to immune defense mechanisms, and the number of LYM and NEU can indicate how well the human body's immune system is functioning [24]. The results showed that compared with the model group, WBC, PLT, LYM, and NEU counts increased after rhCNB treatment, indicating that rhCNB can increase the WBC, PLT, LYM, and NEU counts in peripheral blood of immunocompromised mice induced by cyclophosphamide. In conclusion, the results suggest that rhCNB can enhance general health, promote the differentiation and proliferation of spleen and thymus lymphocytes, and influence immunological function. The peripheral blood picture results reveal that rhCNB can improve the cyclophosphamide-caused reduction in white blood cells, lymphocytes, neutrophils, and platelets. All these suggest that rhCNB can promote the production of immune cells in the body with low immune function, thus enhancing the immune function. By suppressing the activity of monocytes and macrophages, cyclophosphamide can prevent T lymphocytes and B lymphocytes from proliferating and differentiating [25–28]. Additionally, cyclophosphamide can affect cytokine production by activating the NF-*κ*B pathway by affecting signaling pathways such as dendritic cells, TLR_2_, and TLR_4_ [29]. The results of flow cytometry showed that rhCNB could upregulate the expressions of CD4^+^, CD8^+^ T, and CD19^+^ B subpopulations in immunocompromised mice, indicating that rhCNB can promote the differentiation and proliferation of T cells and B cells in immunocompromised mice and regulate and improve the immune response. These results were consistent with the fact that rhCNB could induce the proliferation of B lymphocytes and thymus lymphocytes around the splenic cap in the model group. To maintain normal cellular and humoral immune functions, Th1 and Th2 cells are in a dynamic equilibrium state under normal conditions [30–32]. These immunomodulatory cytokines are involved in T cell proliferation, differentiation, activation, and immunomodulation. IFN-*γ* and IL-4, specific cytokines of Th1 and Th2 cells, respectively, can control the activity of downstream effector cells through Th1 and Th2 cells [33]. IFN-*γ* promotes the differentiation of Th1 cells by activating antigen-presenting cells and upregulating transcription factor T-bet. IL-4 is also a key regulator of the immune response, which can promote the transformation of immature T cells into Th2 cells [34]. Transcription factors T-bet and GATA_3_ are the key factors for TH0 cells to differentiate into Th1 or Th2 cells and for maintaining Th1/Th2 homeostasis [35, 36]. The results showed that the levels of IL-4 and IFN-*γ* in the model group decreased and increased in the treatment group after rhCNB treatment. At the same time, the mRNA levels of T-bet and GATA_3_ decreased in the model group and increased in the treatment group after rhCNB treatment. The results showed that rhCNB could regulate the above cytokine secretion pathways, hence regulating the body's immune response.

As a new anti-tumor protein drug, rhCNB can activate the innate immune system through the TLR4/NF-*κ*B signaling pathway, promote the maturation of dendritic cells, upregulate the expression of macrophages, and induce tumor cell apoptosis [4]. MAPK, a downstream signaling pathway of TLRs, is an important molecular pathway that regulates immune response and is important for T cell proliferation, differentiation, and other related functions. The experimental results found that rhCNB could increase the mRNA levels of TLR_4_, P38, JNK, and ERK in mice with cyclophosphamide-induced immunodeficiency. MAPKs can activate the immune response in T lymphocytes [37]. P38, ERK, and JNK are three pathways in the kinase family. Activated by mitogens and growth factors, ERK can regulate the proliferation and differentiation of Th2 cells [38]. JNK regulates the proliferation, differentiation, and associated cytokine production of CD4+ T cells [39, 40]. P38 mainly conducts inflammatory cytokines and various types of cell stress signals. Recent studies have found that the P38 MAPK signaling may promote the proliferation of T lymphocytes. Also, the activation of the p38MAPK/ERK1/2 signaling pathway can activate B cells, induce the synthesis and secretion of cytokines, and regulate inflammation and immune [41, 42]. In conclusion, rhCNB may stimulate T lymphocyte proliferation and cytokine production through the TLR_4_/MAPK signal pathway, promote the differentiation and proliferation of relevant immune cells, and regulate the body's humoral and cellular immune functions. In addition, rhCNB could activate macrophages and promote dendritic maturation through the TLR_4_ pathway, enhance antigen presentation of antigen-presenting cells, regulate cytokine secretion, and enhance immune response.

## 5. Conclusion

In summary, rhCNB can effectively improve the immunodeficiency induced by cyclophosphamide, enhance the immune response of the body by enhancing the function of immune organs and the phagocytosis of macrophages, and regulate Th1 and Th2 through the TLR_4_/MAPK signal pathway. The secretion of cytokines promotes the differentiation and proliferation of T lymphocytes and B lymphocytes, regulates the balance of Th1/Th2, and finally boosts the immunity of immunocompromised mice.

## Figures and Tables

**Table 1:** Primers used for the RT-PCR study.

Gene name	Forward	Reverse
GAPDH	TGGGTGTGAACCATGAGAAG	GCTAAGCAGCAGTTGGTGGTGC
TLR_4_	GCCATCATTATGAGTGCCAATT	AGGGATAAGAACGCTGAGAATT
P38	AGGAATTCAATGACGTGTACCT	AGGTCCCTGTGAATTATGTCAG
JNK	CCAGCCTTCAGATGCAGCAGTAAG	GGTGTGCTCAGTGGACATGGATG
ERK	ATCTCAACAAAGTTCGAGTTGC	GTCTGAAGCGCAGTAAGATTTT
T-bet	GATCACTCAGCTGAAAATCGAC	AGGCTGTGAGATCATATCCTTG
GATA_3_	ATTACCACCTATCCGCCCTAT	CGGTTCTGCCCATTCATTTTAT

## Data Availability

The datasets used/analyzed in the current study are available from the corresponding author on reasonable request.
